# Momilactones A and B Are α-Amylase and α-Glucosidase Inhibitors

**DOI:** 10.3390/molecules24030482

**Published:** 2019-01-29

**Authors:** Nguyen Van Quan, Hoang-Dung Tran, Tran Dang Xuan, Ateeque Ahmad, Tran Dang Dat, Tran Dang Khanh, Rolf Teschke

**Affiliations:** 1 Division of Development Technology, Graduate School for International Development and Cooperation (IDEC), Hiroshima University, Higashi Hiroshima 739-8529, Japan; nguyenquan26@gmail.com; 2 Department of Biotechnology, NTT Institute of Hi-Technology, Nguyen Tat Thanh University, 298A-300A Nguyen Tat Thanh Street, Ward 13, District 4, Ho Chi Minh City 72820, Vietnam; thdung@ntt.edu.vn; 3 Central Institute of Medicinal and Aromatic Plants, Process Chemistry and Technology Department, Lucknow 226016, India; ateeque97@gmail.com; 4 Khai Xuan International Co., Ltd., 22, 9/53/8 Quan Hoa, Cau Giay District, Hanoi 123000, Vietnam; khaixuan.study@gmail.com; 5 Agricultural Genetics Institute, Pham Van Dong Street, Hanoi 122000, Vietnam; tdkhanh@vaas.vn; 6 Center for Expert, Vietnam National University of Agriculture, Hanoi 131000, Vietnam; 7 Department of Internal Medicine II, Division of Gastroenterology and Hepatology, Klinikum Hanau, 63450 Hanau, Germany; rolf.teschke@gmx.de

**Keywords:** momilactone A, momilactone B, rice grain, rice by-products, anti-diabetes, α-amylase, α-glucosidase, inhibitory activity

## Abstract

Momilactones A (MA) and B (MB) are the active phytoalexins and allelochemicals in rice. In this study, MA and MB were purified from rice husk of *Oryza sativa* cv. Koshihikari by column chromatography, and purification was confirmed by high-performance liquid chromatography, thin-layer chromatography, gas chromatography-mass spectrometry, liquid chromatography-electrospray ionization-mass spectrometry (LC-ESI-MS), and ^1^H and ^13^C nuclear magnetic resonance analyses. By in vitro assays, both MA and MB exerted potent inhibition on α-amylase and α-glucosidase activities. The inhibitory effect of MB on these two key enzymes was greater than that of MA. Both MA and MB exerted greater α-glucosidase suppression as compared to that of the commercial diabetic inhibitor acarbose. Quantities of MA and MB in rice grain were 2.07 ± 0.01 and 1.06 ± 0.01 µg/dry weight (DW), respectively. This study was the first to confirm the presence of MA and MB in refined rice grain and reported the α-amylase and α-glucosidase inhibitory activity of the two compounds. The improved protocol of LC-ESI-MS in this research was simple and effective to detect and isolate MA and MB in rice organs.

## 1. Introduction

Diabetes has become a worldwide health problem in developed and developing countries. It is reported that about 425 million people are suffering from diabetes which accounts for 12% of global health expenditure [1]. Among diabetic types, type 2 is the most common occurrence in adults due to a complex metabolic abnormality including insulin resistance, hyperglycemia, and deficient insulin secretion. The pathogenesis of type 2 diabetes may launch from many factors such as genetic predisposition, environment, and pancreatic beta-cell dysfunction [2]. One of the most beneficial therapeutics was proposed as maintaining blood glucose at normal levels after a meal [3]. This approach may gradually help avoid chronic hyperglycemia, reduce insulin resistance and consequently stabilize the insulin production of pancreatic beta-cell. In humans, the digestion of carbohydrates is carried out by multiple hydrolytic enzymes. Of which, α-amylase and α-glucosidase are crucial in the hydrolysis of polysaccharides to obtain glucose more suitable for absorption [4]. Hyperglycemia in patients with type 2 diabetes was attributed to starch breakdown by pancreatic α-amylase and glucose uptake by intestinal α-glucosidase [5]. In fact, there are many available antidiabetic drugs such as biguanides, sulfonylureas, meglitinides, thiazolidinediones, α-glucosidase inhibitors, incretin mimetics, dipeptidyl peptidase-IV inhibitors and insulin; however, the use of these pharmaceutical drugs may cause undesired and severe side effects [6]. Therefore, the search for and discovery of new α-amylase and α-glucosidase inhibitors from natural sources are valuable approaches to more securely decelerating the glucose production in the human intestine.

**Figure 1:**
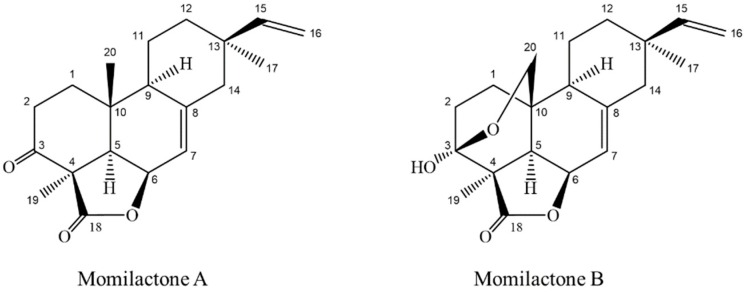
Structures of momilactone A and momilactone B [6,7].

Momilactones A (MA) and B (MB) belonging to diterpenes group (Figure 1), have been detected only in rice and the moss *Hypnum plumaeforme* [7,8]. The allelopathic function of MA and MB against plant’s natural enemies such as weeds and blast fungus has widely been reported [9,10,11,12,13,14]. Recently, MA and MB were found more implicative with salinity and drought tolerance of rice more than allelopathy [15,16]. MA and MB also exhibited antioxidant [14], cytotoxic [17], antitumor [18] and anticancer activities [19,20]. Of which, MB was in lower quantity in rice husk and other plant parts but exerted greater biological activities than MA [21,22]. In addition, Kang et al. [22] showed that MB was effective in controlling ketosis associated with low blood sugar levels, however, trials in hyperglycemic conditions have not been performed. Hitherto, the search of natural compounds with potent anti-diabetes properties has been expanded, but none of the compounds with diterpene lactone structure possessing antidiabetic property was reported, except eremanthin (a sesquiterpene lactone) and andrographolide (a diterpenoid lactone). Among these, eremanthin exhibited hypoglycemic and hypolipidemic activities [23], while andrographolide was potent for diabetic control [24,25,26,27]. There were several other reports on antidiabetic activity of diterpenes and their synthetic derivatives [28,29,30,31,32].

Moreover, no reports on toxicities of natural compounds from either rice grain or its by-products affecting human health have been published. Several compounds involved in the diabetic inhibition were found in rice bran and color rice [33,34,35,36]. Though MA and MB are promising bioactive constituents in rice, the isolation and purification of MA and MB are complicated and laborious. At present, there are very few laboratories in the world that can successfully isolate and purify MA and MB. As a result, no commercial MA and MB from chemical companies in Japan or abroad can be purchased; thus, research on biological activities of the two compounds has been limited. We recently developed a new protocol for extracting conditions and solvents to provide optimal yields of MA and MB by column chromatography combined with different extracting solvents and temperature [8,37]. In this study, we investigated the inhibition of MA and MB on α-amylase and α-glucosidase activities and reported the presence of the two compounds in white rice grain using LC-ESI-MS technique.

## 2. Results

### 2.1. Isolation and Confirmation of Momilactones A and B

#### 2.1.1. HPLC

**Figure 2:**
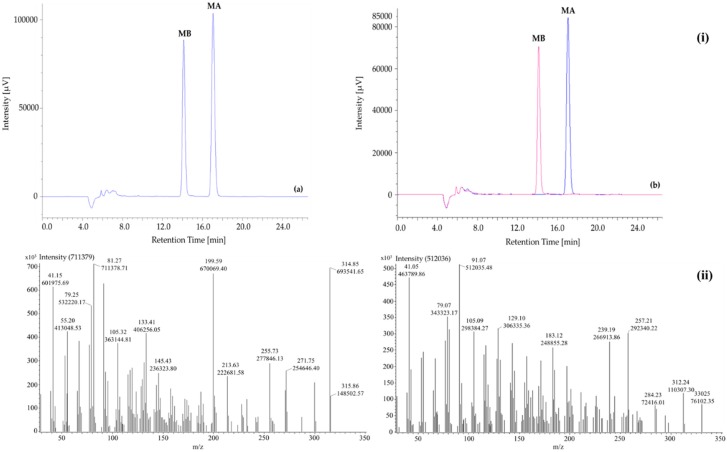
(**i**) HPLC chromatograms of momilactones A and B: (**a**) mixture of standard momilactones A (MA) and B (MB), (**b**) isolated momilactones A and B (overlaid chromatogram); and (**ii**) Mass spectra of the purified (**a**) momilactone A and (**b**) momilactone B.

By an open column chromatography with chloroform as mobile phase, two compounds were purified including MA (52 mg) and MB (44 mg). The presence of MA and MB was confirmed by HPLC at 210 nm spectra (Figure 2). The peaks were affirmed by measuring a mixture of standards and the isolated MA and MB at ratio 1:1 (data not presented). According to Figure 2i, the isolated MA and MB appeared at 17.03 ± 0.02 min and 14.06 ± 0.01 min, respectively. The separation order was in accordance with standard MA (17.03 ± 0.03 min) and MB (14.06 ± 0.02 min). The retention times were also coincident with those reported in previous research [21,22,38,39]. Detection limits of MA and MB were 0.43 and 0.18 ng/mL, respectively. Meanwhile, limits of quantitation were calculated as 1.31 ng/mL for MA and 0.54 ng/mL for MB.

#### 2.1.2. GC-MS

The mass spectral data of the isolated MA and MB were shown in Figure 2ii. The earlier detected peak (retention time = 23.50 min) showed a molecular ion at 315.86 *m*/*z* while this value of later one (retention time = 23.70 min) was 330.25 *m*/*z*. There were 161 and 165 fragments of MA and MB were obtained, respectively. The fragmentation patterns (161 and 165 for MA and MB, respectively) and intensity data of the two compounds were presented in the Supplementary Materials, Tables S1 and S2.

#### 2.1.3. ^1^H-NMR and ^13^C-NMR

MA: Colourless crystalline compound; R_f_ 0.71 (CHCl_3_:MeOH; 9.5:0.5); m.p. 234–236 °C; IR ν_max_: 2936, 1766, 1698, 1637, 1390, 1188, 990, 908; ^1^H-NMR (CDCl_3_; 500 MHz): *δ* 1.90 (m, H_2_-1α), 2.59–2.63 (m, H-2), 2.31 (d, *J* = 5.0, H-5), 4.84 (t, *J* = 5.0, H-6), 5.70 (d, *J* = 5.0, H-7), 1.74–1.80 (m, H-9, H-11α), 1.32 (m, H_2_-11β), 1.56–1.62 (m, complex, H_2_-1β, H_2_-12), 2.20, 2.19 (d, *J* = 12.5, H_2_-14), 5.84 (d, *J* = 17.0, 11.0, H-15), 4.97, 4.93 (d, *J* = 17.0 & 1; 10.0 & 1, H-16), 0.88 (s, H-17), 1.52 (s, H-18), 0.98 (s, H-20). ^13^C-NMR (CDCl_3_; 125 MHz): *δ* 34.89 (C-1), 31.21 (C-2), 205.20 (C-3), 53.57 (C-4),46.46 (C-5), 73.17 (C-6), 114.03 (C-7), 148.96 (C-8), 50.18 (C-9), 32.46 (C-10), 23.99 (C-11), 37.24 (C-12), 40.13 (C-13), 47.53 (C-14), 148.03 (C-15), 110.17 (C-16), 21.80 (C-17), 21.47(C-18), 174.32 (C-19), 21.96 (C-20); HPLC-PDA-MS ESI^+^: 315 [M + H]^+^ (C_20_H_27_O_3_); ESI^−^: 313 [M − H]^−^ (C_20_H_25_O_3_); HRMS 315.1959 [M + H]^+^ (calc for C_20_H_27_O_3_, 315.1960). (Compare NMR data with previous literature [8,12,18]).

MB: Colourless crystalline compound; R_f_ 0.63 (CHCl_3_:MeOH; 9.5:0.5); m.p. 240 °C; IR ν_max_: 2920, 1737, 1662, 1637, 1461, 1296, 992, 916; ^1^H-NMR (CDCl_3_; 500 MHz): *δ* 1.99 (m, H-1α), 2.13–2.06 (m, complex H-2, H-14), 2.20 (dd, *J* = 6.5, 2.0, H-5), 4.97 (t, *J* = 4.5, H-6), 5.68 (d, *J* = 5.0, H-7), 1.72–1.64 (m, H-9, H-11α), 1.30 (m, H-11β), 1.56–1.51 (m, complex, H-1β, H-12), 5.82 (dd, *J* = 17.0, 11.0, H-15), 4.93 (d d, *J* = 10.0 & 1, H-16), 0.87 (s, H-17), 1.43 (s, H-18), 3.58, 4.07 (dd, 9.0, 3.1 7 9.0, 3.5). ^13^C-NMR (CDCl_3_; 125 MHz): *δ* 28.81 (C-1), 26.44 (C-2), 96.60 (C-3), 50.35 (C-4), 42.97 (C-5), 73.76 (C-6), 114.00 (C-7), 146.70 (C-8), 44.68 (C-9), 30.74 (C-10), 24.79 (C-11), 37.22 (C-12), 39.99 (C-13), 47.42 (C-14), 148.83 (C-15), 110.23 (C-16), 21.86 (C-17), 18.99 (C-18), 180.48 (C-19), 72.72 (C-20); HPLC-PDA-MS ESI^+^: 331 [M + H]^+^ (C_20_H_27_O_4_); ESI^−^: 329 [M − H]^−^ (C_20_H_25_O_4_); HRMS 330.1905 [M + H]^+^ (calc for C_20_H_27_O_4_, 331.1909). (Compare NMR data with previous literature [8,12,18]).

### 2.2. In Vitro Inhibition of α-Amylase and α-Glucosidase

To the best of our knowledge, no study has before examined the inhibitory activities of MA and MB on the two key enzymes α-amylase and α-glucosidase which are relevant to diabetes. The inhibition of MA and MB was positively correlated with their concentrations (r^2^ = 0.94 and 0.80 for α-amylase; r^2^ = 0.83 and 0.95 for α-glucosidase, respectively) (Supplementary Materials, Figures S1 and S2). The IC_50_ values were presented in Table 1. Accordingly, both MA and MB exerted inhibitory activity against α-amylase (IC_50_ = 266.68 ± 2.74 and 146.85 ± 1.95 µg/mL, respectively) and α-glucosidase (IC_50_ = 991.95 ± 0.96 and 612.03 ± 0.39 µg/mL, respectively). These effects were also in line with those of the well-known commercial diabetes inhibitors (acarbose: IC_50_ = 117.08 ± 1.47 µg/mL for α-amylase and 2549.00 ± 5.15 µg/mL for α-glucosidase; quercetin: IC_50_ = 105.68 ± 0.09 µg/mL for α-glucosidase). Both MA and MB and acarbose exhibited stronger inhibition on α-amylase than on α-glucosidase activities. By comparing the IC_50_ values, it was clear that MA and MB have levels of antidiabetic capacity similar to acarbose. More specifically, MA and MB even exhibited much stronger suppressive properties than acarbose on α-glucosidase inhibitory assay, for which MA was 2.5-fold and MB was 4.2-fold greater than acarbose.

### 2.3. Contents of MA and MB in Rice Plant Parts

Results from a two-way ANOVA analysis (Table 2) revealed that there was a significant difference between MA and MB contents. Furthermore, the momilactone contents significantly varied among rice plant organs. Additionally, interaction value represented the mismatch between MA and MB contents in various rice parts. In particular, MA was detected in a higher amount than MB in grain, husk, and root, but in contrast, the content was lower in leaf (*p* < 0.05). The highest MA quantity was in husk (16.44 ± 0.09 µg/g DW), whereas the maximum MB content was found in leaf (12.73 ± 0.36 µg/g DW) as compared with other plant parts (Table 2). Because both MA and MB were antidiabetic chemicals, rice grain and different plant parts of rice might be useful to exploit for antidiabetic treatment. This finding was in line with previous reports that in rice organs, the amount of MA was higher than MB [15,21,22,38].

**Figure 3:**
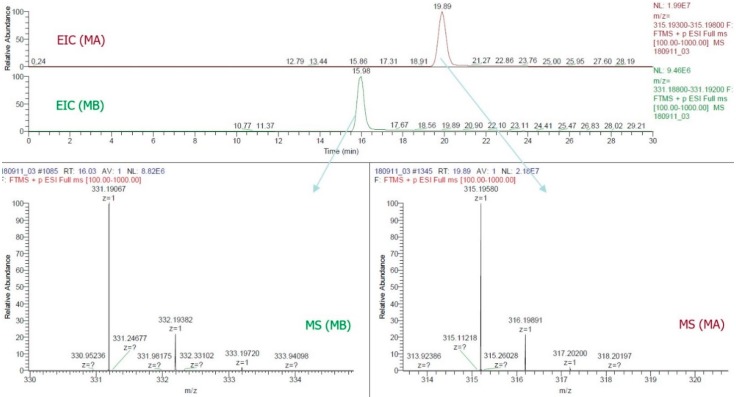
Extracted ion chromatograms and mass spectra of standard momilactones A and B.

**Figure 4:**
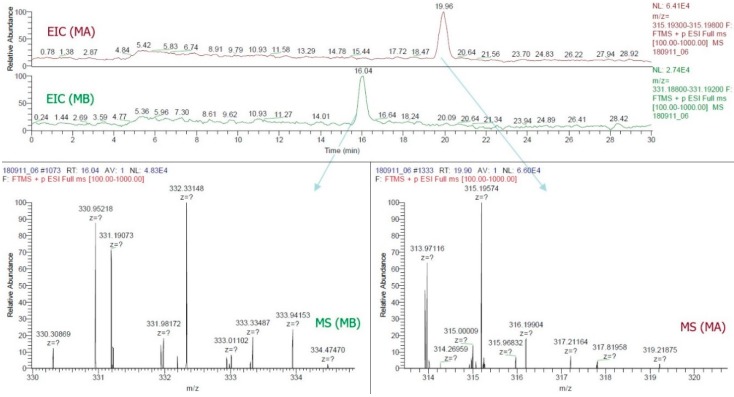
Extracted ion chromatograms and mass spectra of momilactones A and B detected in rice grain.

By this study, the LC-ESI-MS method was the first to confirm the presence of MA and MB in rice grain. The use of a positive FTMS mode, mass range scanned from *m*/*z* = 315.193−315.198 and *m*/*z* = 331.188−331.192, the EIC of all samples (Figure 3) provided two major peaks which were finally confirmed as MA (RT~19.89, C_20_H_27_O_3_, 315.196) and MB (RT~16.03, C_20_H_27_O_4_, 331.190). Figure 4 illustrated the LC-ESI-MS results which confirmed the presence of the standards MA and MB in rice grain sample by analyzing the similarities of retention time integrated with fragmentation patterns. Mass spectra data of grain sample showed that patterns at 315.19574 and 331.19073 *m*/*z* entirely matched with standard patterns at 315.19580 for MA and 331.19073 for MB (Figure 3 and Figure 4).

## 3. Discussion

In this study and for the first time, we revealed that MA and MB (diterpene lactones) are found in white rice grain and exhibited potential inhibitory effects on α-amylase and α-glucosidase, two key enzymes relevant to type 2 diabetes [5]. MA and MB were principally found in rice husk [10,21,22,38], leaf [15], root and root exudates [7,10]. The content and presence of MA and MB varied among rice cultivars and growing stage [15]. The present research highlighted the promising antidiabetic activity of MA and MB, detected and quantified the two compounds in rice grain. Individually, MB displayed a stronger inhibitory activity than MA on both α-amylase (1.8-fold) and α-glucosidase (1.6-fold) (Table 1). The presence of a lactone ring in chemical structure has been reported to play a major role for α-glucosidase suppression [40]. On the other hand, the presence of a hydroxyl group in the diterpenoid part might explain the difference in bioactivity between MA and MB. Particularly, the hydroxyl group at C-3 in the structure of MB could increase the antidiabetic competence as compared to MA (Figure 1). The examination of functional groups in bioactive compounds is important in order to develop novel synthesized derivatives, which possess a stronger activity and are more economical than their parent compounds. For instance, DDK and DK are the two kavalactones in *Alpinia zerumbet*, a plant that has been demonstrated to play a potential role in longevity of Japanese living in one of the southernmost islands of the Ryukyus, Japan [41]. Accordingly, many derivatives of DDK and DK have been synthesized and show promising medicinal and pharmaceutical properties [42,43,44]. Further elaboration on functional chemical groups in the diterpene lactone structures of MA and MB should be conducted to be compared in terms of their potential inhibition on diabetes both in vitro models and in human randomized clinical trials. In addition, other potent antidiabetic compounds in rice grain might be further identified by using the advanced techniques from the LC-ESI-MS protocol developed by this study.

There were sporadic studies to examine the diabetes inhibitory potential of rice husk and straw, but they sorely investigated activity of the complete rice extracts. Yang et al. [45] demonstrated the effect of liquid rice hull smoke extract against diabetes using an in vivo model, but the chemical composition of the extract was not mentioned. Yehia and Saleh [46] only noted the inhibitory effect of rice straw extract on amylase activity of some fungi without successfully identifying bioactive substances as well as antidiabetic competency. Heretofore, the digestion of white or refined rice grain has been thought to be positive on diabetes occurrence, as rice grain is rich in carbohydrates [47,48]. Therefore, diabetic patients are often advised to ingest as low as possible white rice grain in their meals or to use other alternative grains such as brown and color rice. Several compounds derived from brown or colored rice grains containing γ-oryzanol and polyphenols such as phenolic acids and anthocyanins were reported to be potent in type 2 diabetes reduction [49,50,51,52,53]. Instead by this study, we hypothesized that the ingestion of rice might be beneficial against diabetes because of the presence of MA and MB which showed the higher inhibitory level on α-glucosidase activity than the commercial diabetes inhibitor acarbose by in vitro assays (Table 1). The contents of 2.07 µg MA and 1.06 µg MB per g dry weight in rice grain (Table 2) were equivalent to 128.8–138.5 and 479.2–577.3 g rice grain that was estimated to provide effective MA and MB on 50% inhibition of α-amylase and α-glucosidase activity, respectively.

Several natural products have been reported to be α-amylase and α-glucosidase inhibitors [40,54,55,56]. However, there was no uniformity in the inhibition results compared with the standard acarbose, which might be due to different sources of tested enzymes. Hence, further assays on human and mammalian enzymes are apparently needed to examine the benefits of MA and MB against diabetes risk. In addition, side effects of MA and MB should be carefully searched for, although no negative report on any natural rice compound is available. Finally, an in vivo model is absolutely needed to confirm the positive effects of MA and MB on insulin content, cardiovascular as well as intestine problems, and absorption processes. Furthermore, the search for other α-amylase and α-glucosidase inhibitors including derivative constituents of MA and MB in rice grain should be further conducted.

Thus far, MA and MB have been known as phytoalexins and allelochemicals in rice leaf, root, husk, and root exudate [7,8,9,10,11,12,13,14,15,16,17]. This present research was the first one to detect the presence of MA and MB as new α-amylase and α-glucosidase inhibitors in rice grain by using the improved protocol of LC-ESI-MS (Figure 3 and Figure 4). It provided detailed mass spectra on detecting of MA and MB in both GC-MS (Figure 2ii) and LC-ESI-MS (Figure 3 and Figure 4). It will thus help achieve more accessible and accurate detection of MA and MB, as well as their potent derivatives in rice grain and rice organs. Although MA and MB showed persuasive α-amylase and α-glucosidase inhibitory activities, more in vivo and clinical trials should confirm the efficacy of MA and MB against diabetes. This observation can estimate the effective amounts of MA and MB in rice grain, thus aiding the breeding of rice cultivars rich with MA and MB.

## 4. Materials and Methods

### 4.1. Collection and Extraction of Rice Husk

Rice husk of Koshihikari variety (Japonica subtype) was collected from rice mills allocated near Hiroshima University, Higashi-Hiroshima Campus, Japan in July 2017. The identification of variety was authenticated by a voucher specimen (KOS-MOMI 17HJ) which was deposited at Plant Physiological Laboratory, IDEC, Hiroshima University, Japan. Extraction and isolation methods were referred from previous studies [37,57] with slight modifications. Briefly, rice husks (7 kg) were dried at 50 °C for 6 days by an oven and steeped in 50 L methanol 100% for 2 weeks at room temperature to yield 92 g crude extract. After mixing with an adequate volume of distilled water, the extract was partitioned consecutively with hexane and ethyl acetate (EtOAc) at high grades (>99.5%).

### 4.2. Isolation of Momilactones A and B from EtOAc Extract

The dry EtOAc extract (57 g) was chromatographed on silica gel (60–100 mesh) column (5 × 60 cm) to yield 35 fractions with the following eluants: 5 fractions in hexane (500 mL/elution), 20 fractions in hexane:EtOAc (9:1) (200 mL/elution), 10 fractions in hexane:EtOAc (8:2) (200 mL/elution). Fractions from hexane:EtOAc (8:2) were combined, evaporated and further purified by column chromatography over silica gel (200–400 mesh) with chloroform. Similarity among fractions was tested by TLC prior to combination [37].

### 4.3. Identification and Confirmation of Momilactones A and B by HPLC, TLC, GC-MS, and ^1^H-NMR and ^13^C-NMR Analyses

The presence and purity of MA and MB by HPLC and TLC analyses were compared with the standards MA and MB attained from a previous study [37]. For HPLC analysis, the isolated MA and MB were separately dissolved in methanol and filtered by a 0.45 μm pore size polytetrafluoroethylene filter (FILTSTAR Syringe Filter, Starlab Scientific Co., Ltd., China). The final concentration of tested samples was adjusted to 20 µg/mL, the standard MA and MB were mixed at ratio 1:1 (v/v). The HPLC system including PU-4180 RHPLC pump, LC-Net II/ADC controller, and UV-4075 UV/Vis detector (Jasco, Tokyo, Japan) equipped with a Waters Spherisorb ODS2 (10 μm, 250 mm × 4.6 mm i.d.) column (Waters Cooperation, Milford, MA, USA). Mobile phase comprised 0.1% trifluoroacetic acid in 70% acetonitrile. The flow rate was adjusted to 0.4 mL/min within 30 min. The detector was set at 210 nm. The injection volume was 10 µL. Data acquisition was executed on ChromNAV software (JASCO, Tokyo, Japan). The sensitivity of HPLC system was determined and expressed as limits of detection (LOD) and limits of quantitation (LOQ) by linear regression analyses of peak areas against concentrations of individual momilactone A and B.

TLC analysis was carried out on the TLC silica gel 60 plates (Merck KGaA, Darmstadt, Germany) with a layer thickness of 175–225 μm. Solution of the standards MA and MB (1:1, v/v) was used as a reference. Specifically, MA and MB were spotted on a TLC plate and run with the solvent system of chloroform:methanol (9.5:0.5, v/v) for 2.4 min. The plate was then dipped into a chamber comprising of 1% vanillin-sulfuric acid in pure ethanol and dried in an oven at 100 °C for 2 min. Subsequently, the separation of MA and MB was observed visually and the retention factor (R_f_) value of each MA and MB was calculated as:

R_f_ = distance of MA or MB spot traveled/distance of solvent system moved (1)

where the molecular mass of the isolated MA and MB was confirmed by spectrum data from GC-MS analysis. The GC-MS system (JMS-T100 GCV, JEOL Ltd., Tokyo, Japan) equipped with an auto sampler coupled with a 30 m × 0.25 mm I.D. × 0.25 μm film thickness DB-5MS column (Agilent Technologies, J & W Scientific Products, Folsom, CA, USA). A concentration of 1000 µg/mL of either isolated momilactones was used. Helium was used as a carrier gas at split ratio 5:1. The GC oven conditions were as follows: the initial temperature was 50 °C without hold time, boosted temperature up to 300 °C at 10 °C/min, and held for 20 min. The injection port and detector temperature were set at 300 °C and 320 °C, respectively. The mass range scanned from 29 to 800 amu. The control of the GC-MS system and the confirmation of analytes were conducted using JEOL’s GC–MS Mass Center System Version 2.65a.

Both ^1^H- and ^13^C-nuclear magnetic resonance (NMR) spectra were achieved on a Brucker DRX-500 model spectrometer (Bruker India Scientific Pvt. Ltd., New Delhi, India) operated at 500 and 125 MHz, respectively. The NMR spectra were received in deuterated CHCl_3_ using tetramethylsilane (TMS) as an internal standard. The fast atom bombardment mass spectroscopy (FABMS) data were recorded on a JEOL SX-102 spectrometer (JEOL USA Inc., Peabody, MA, USA) and electrospray ionization mass (ESI) in direct mass analysis of high performance liquid chromatography-photodiode array-mass spectrometry detectors (HPLC-PDA-MS) spectrometer (Shimadzu Corporation, Kyoto, Japan) and high-resolution mass spectrometry (HRMS) was measured on Agilent technology 6545Q-TOF LC/MS (5301 Stevens Dreek Blvd. Santa Clara, CA, USA). Infrared spectroscopy was recorded on a fourier transform infrared (FT-IR) spectrophotometer Shimadzu 8201 PC (4000–400 cm^−1^) (Shimadzu Cooporation, Kyoto, Japan).

### 4.4. α-Amylase Inhibition Assay

The inhibitory effect of MA and MB on α-amylase was assessed by starch-iodine method [4] with a modified model as follows: in each well of a microplate (U-shape, Greiner Bio-one, NC, USA), 20 µL of each either MA or MB were pre-incubated with 20 µL of 1 U/mL α-amylase solution (from *Aspergillus oryzae*, Sigma-Aldrich, St. Louis, MO, USA) at 37 °C for 10 min. The reaction was initiated by pipetting 30 µL of starch (0.5% in deionized water) (soluble ACS reagent, Sigma-Aldrich, St. Louis, MO, USA). After 6 min incubation at 37 °C, an aliquot of 20 µL of hydrochloric acid (1 M) were added to stop reaction, followed by 120 µL of 0.25 mM aqueous iodine solution. The absorbance at 565 nm was read by a microplate reader (Multiskan^TM^ Microplate Spectrophotometer, Thermo Fisher Scientific, Osaka, Japan). The inhibitory activity of MA and MB on α-amylase was performed as the inhibition percentage that was calculated by the following formula:

% inhibition = (A − C)/(B − C) × 100(2)

where A is the absorbance of reaction with presence of MA or MB, B is the absorbance of reaction without enzyme, C is the absorbance of reaction with absence of the momilactones. A commercial diabetes inhibitor acarbose was used as a positive reference. Dilutions of test samples and dissolutions of enzyme used 20 mM sodium phosphate buffer (pH 6.9 comprising of 6 mM sodium chloride). α-Amylase solution and soluble starch solution were prepared and used on the day of experiment. The IC_50_ value was calculated to exhibit 50% inhibitory capacity of reaction at a certain concentration. Therefore, lower value of IC_50_ indicates stronger activity.

### 4.5. α-Glucosidase Inhibition Assay

The anti-α-glucosidase activity of MA and MB was evaluated using a method as described previously [58] with some alterations. In brief, an amount of 20 µL methanolic stock solution of MA and MB were pre-mixed with an equal volume of 0.1 M potassium phosphate buffer (pH 7) and 40 µL of α-glucosidase (from *Saccharomyces cerevisiae*, Sigma-Aldrich, St. Louis, MO, USA) enzyme solution (0.5 U/mL in 0.1 M potassium phosphate buffer, pH 7). After 6 min incubation at 25 °C, a 20 µL aliquot of 5 mM *p*-nitrophenyl-α-D-glucopyranoside (pNPG) substrate (in 0.1 M potassium phosphate buffer, pH 7) was added to each reaction and the mixture was incubated for another 8 min. Eventually, the reaction was terminated by adding 100 µL of 0.2 M Na_2_CO_3_, and absorbance was recorded at 405 nm. The inhibition percentage was calculated by the following equation:

% inhibition = (1 − A_sample_/A_control_) × 100 (3)

where A_sample_ is absorbance of reaction with either MA or MB or positive controls (acarbose or quercetin) and A_control_ is absorbance of reaction with 20% methanol as a negative control. IC_50_ value was obtained by the same way as above.

### 4.6. Quantification and Confirmation of MA and MB in Rice Plant Parts by HPLC and LC-ESI-MS

Rice grain, husk, leaf and root of Koshihikari rice variety were collected in July–October 2018, Higashi Hiroshima, Japan. Samples were pre-soaked into NaOCl 0.5% for 15 min, then cleaned several times by distilled water. After blotting, they were dried by an oven at 50 °C for 6 days. Dried samples were separately extracted by MeOH for 5 days. Subsequently, each crude methanolic extract was obtained and mixed with an equal volume of hexane in a separatory funnel. After 2 h, the lower phase of every extraction was collected, filtered, and concentrated to yield a concentration of 100 mg/mL. HPLC analysis was conducted in a similar method as described above. The identification and quantification of MA and MB in rice plant parts were compared and calculated according to the retention times and peaks of the standards MA and MB with the samples. LC-ESI-MS analysis was implemented by using an LTQ Orbitrap XL mass spectrometer (Thermo Fisher Scientific, CA, USA) connected with an electrospray ionization (ESI) source. LC condition was set in the same way with HPLC analysis. ESI condition comprised: ion spray voltage was 4.5 kV, sheath gas flow rate was 60 and aux gas flow rate was 20. MS analysis was run by a positive fourier transform mass spectrometer (FTMS) at a resolution of 60,000 with a scan range of *m*/*z* 100–1000. 10 µL of samples (50 mg/mL) and standard momilactones (0.5 mg/mL) were injected to the above system by an autosampler. The presence of momilactones in samples was confirmed by comparing their extracted ion chromatograms (EIC) and mass spectra with those of standard momilactones.

### 4.7. Statistical Analysis

Data of this study were elaborated on the Minitab 16.0 software (Minitab Inc., State College, PA, USA). All analyses were in a complete randomization with three replications, and results are displayed as mean ± standard error (SE). Significant differences were determined by one-way and two-way ANOVA using Tukey’s test at *p* < 0.05.

## 5. Conclusions

By in vitro assays and for the first time, the present study discovered that MA and MB detected in and isolated from rice were effective inhibitors of α-amylase and α-glucosidase, which may be explored as novel and potent candidates for antidiabetic therapy. However, further studies should be implemented to assert applicable doses of MA and MB before conducting medicinal production, pre-clinical and clinical trials on the two compounds. The identification and quantification of MA and MB in refined rice grain by the HPLC-ESI-MS technique were the principal findings of this study.

## Figures and Tables

**Table 1:** α-Amylase and α-glucosidase inhibitory activities of Momilactones A (MA) and Momilactones B (MB).

	α-Amylase Inhibitory Assay (µg/mL) (IC_50_)	α-Glucosidase Inhibitory Assay (µg/mL) (IC_50_)
MA	266.68 ± 1.58 ^c^	991.95 ± 0.96 ^c^
MB	146.85 ± 1.12 ^b^	612.03 ± 0.39 ^b^
Acarbose	117.08 ± 0.85 ^a^	2549.00 ± 5.15 ^d^
Quercetin	-	105.68 ± 0.09 ^a^

Data presented means ± standard errors. Means within a column followed by different superscript letters (^a, b, c, d^) are significantly different at *p* < 0.05 level. -, not measured; MA, momilactone A; MB, momilactone B.

**Table 2:** Momilactone contents in rice grain, husk, leaf, and root (µg/g DW).

Rice Organs	Momilactone A	Momilactone B
Grain	2.07 ± 0.01 ^d^	1.06 ± 0.01 ^d^
Husk	16.44 ± 0.09 ^a^	9.24 ± 0.04 ^b^
Leaf	4.28 ± 0.03 ^c^	12.73 ± 0.36 ^a^
Root	8.06 ± 0.13 ^b^	5.69 ± 0.19 ^c^
**Two-Way ANOVA**		
Momilactones	*	*
Rice organs	*	*
Interaction	*	*

Data presented means ± standard errors. Means within a column followed by different superscript letters (^a, b, c, d^) are significantly different; and * indicates a significant difference at *p* < 0.05.

## Supplementary Materials

The provided Tables S1 and S2 are available online.
