# Supplementary material for: Momilactones A and B Are α-Amylase and α-Glucosidase Inhibitors
Source: Molecules. 2019 Jan 29;24(3):482. doi: 10.3390/molecules24030482 (PMC6385104; doi:10.3390/molecules24030482)
Supplement: Supplementary file 1 [file molecules-24-00482-s001.pdf]

Table S1. The fragmentation patterns and intensity data of momilactone A

Peak	Fragmentation pattern (m/z)	Relative intensity (%)	Intensity	Peak	Fragmentation pattern (m/z)	Relative intensity (%)	Intensity
1	29.13	21.92	155949.10	82	136.40	7.08	50387.20
2	39.13	23.86	169761.76	83	137.38	8.58	61067.64
3	40.14	7.56	53771.94	84	141.40	13.24	94220.79
4	41.15	84.62	601975.69	85	142.40	11.43	81336.00
5	42.13	4.31	30639.69	86	143.41	27.59	196281.84
6	42.16	6.10	43422.10	87	144.42	12.24	87041.62
7	43.14	14.86	105744.85	88	145.43	33.22	236323.80
8	43.17	11.21	79721.02	89	146.44	12.74	90649.82
9	44.11	2.01	14318.28	90	147.45	19.91	141634.95
10	51.16	6.01	42770.76	91	148.43	8.28	58871.09
11	52.17	3.89	27673.41	92	149.44	8.29	58947.08
12	53.18	45.10	320831.78	93	150.41	6.99	49701.97
13	54.19	5.71	40602.19	94	151.42	6.96	49543.00
14	55.16	22.41	159393.50	95	152.42	4.87	34639.45
15	55.20	58.06	413048.53	96	153.42	5.16	36676.51
16	56.21	3.07	21815.81	97	154.43	3.39	24138.81
17	57.18	3.51	24982.46	98	155.44	11.07	78765.00
18	57.22	2.88	20512.19	99	156.45	7.79	55396.87
19	65.21	23.86	169748.99	100	157.46	23.95	170372.08
20	66.22	9.82	69825.32	101	158.47	9.80	69738.01
21	67.23	53.80	382724.15	102	159.48	20.95	149038.77
22	68.20	3.77	26821.67	103	160.45	3.73	26568.96
23	68.23	14.37	102248.88	104	160.49	5.71	40653.62
24	69.21	8.06	57363.93	105	161.46	15.08	107279.19
25	69.25	10.81	76901.74	106	162.47	5.33	37898.79
26	77.23	51.52	366475.96	107	162.50	3.17	22559.26
27	78.24	13.64	97064.75	108	163.48	6.51	46286.97
28	79.25	74.82	532220.17	109	165.45	3.60	25641.83
29	80.26	14.85	105606.64	110	167.46	3.15	22439.70
30	81.27	100.00	711378.71	111	169.48	10.26	72961.77
31	82.28	8.63	61374.82	112	170.49	5.96	42416.07
32	83.22	4.92	35019.92	113	171.50	19.28	137173.16
33	83.25	3.03	21585.51	114	172.51	7.36	52340.88
34	83.29	3.49	24802.88	115	173.49	18.60	132304.97
35	89.26	2.43	17303.54	116	174.49	8.66	61583.81
36	91.28	88.12	626895.60	117	175.50	15.45	109879.62
37	92.28	13.45	95648.47	118	176.51	6.21	44146.60
38	93.30	35.37	251644.15	119	177.49	10.92	77678.72
39	94.30	9.00	64016.23	120	183.53	9.04	64324.66
40	95.28	5.16	36724.36	121	184.53	4.87	34633.34
41	95.32	29.91	212803.54	122	185.55	15.22	108288.25
42	96.32	2.69	19162.76	123	186.52	2.68	19061.10

Peak	Fragmentation pattern (m/z)	Relative intensity (%)	Intensity	Peak	Fragmentation pattern (m/z)	Relative intensity (%)	Intensity
43	97.26	2.77	19708.39	124	186.55	4.27	30381.33
44	97.30	3.20	22740.45	125	187.53	21.93	156017.39
45	102.29	2.44	17378.63	126	188.54	9.51	67646.72
46	103.30	13.14	93478.42	127	189.55	16.48	117207.09
47	104.31	6.43	45713.91	128	190.52	3.26	23175.01
48	105.32	51.05	363144.81	129	190.56	5.19	36913.76
49	106.33	12.80	91061.47	130	192.51	3.51	24967.78
50	107.30	3.66	26061.82	131	197.57	4.24	30190.81
51	107.34	20.50	145854.91	132	198.58	5.08	36143.67
52	108.31	4.27	30361.90	133	199.59	94.19	670069.40
53	108.35	5.29	37632.60	134	200.58	21.03	149620.70
54	109.32	9.10	64745.74	135	201.57	14.38	102311.84
55	109.36	4.24	30151.68	136	202.58	11.09	78896.30
56	110.33	4.32	30766.11	137	213.63	31.30	222681.58
57	111.31	3.93	27947.45	138	214.64	9.36	66613.44
58	115.33	33.93	241361.78	139	217.56	5.92	42078.07
59	116.34	14.78	105139.90	140	223.64	3.68	26193.83
60	117.35	36.34	258546.65	141	227.66	16.17	115022.23
61	118.35	12.89	91694.09	142	228.66	9.42	67046.42
62	119.37	37.97	270075.96	143	229.66	8.28	58922.01
63	120.37	10.58	75287.47	144	232.61	17.62	125323.91
64	121.35	8.16	58065.78	145	233.62	3.08	21931.57
65	121.38	10.20	72560.31	146	241.68	8.12	57779.21
66	122.36	8.17	58129.18	147	242.69	5.32	37847.55
67	122.39	2.81	19994.57	148	243.70	8.53	60673.48
68	123.37	23.63	168081.69	149	255.73	39.06	277846.13
69	124.38	10.02	71312.31	150	256.73	7.80	55473.33
70	127.35	7.49	53285.42	151	257.71	6.31	44893.35
71	128.36	26.81	190715.16	152	258.72	5.74	40835.30
72	129.37	30.94	220112.47	153	259.73	4.28	30431.47
73	130.38	12.58	89459.41	154	270.78	24.40	173608.38
74	131.39	41.00	291634.31	155	271.75	35.80	254646.40
75	132.40	16.81	119599.81	156	272.76	11.55	82142.84
76	133.41	57.11	406256.05	157	286.80	8.47	60244.49
77	134.38	2.82	20029.66	158	299.80	29.08	206867.80
78	134.42	10.48	74535.18	159	300.81	6.01	42747.39
79	135.39	6.34	45075.48	160	314.85	97.49	693541.65
80	135.43	6.92	49245.99	161	315.86	20.88	148502.57
81	136.37	7.70	54755.48				

Table S2. The fragmentation patterns and intensity data of momilactone B

Peak	Fragmentation pattern (m/z)	Relative intensity (%)	Intensity	Peak	Fragmentation pattern (m/z)	Relative intensity (%)	Intensity
1	29.01	2.89	14790.27	84	154.11	7.11	36391.31
2	29.05	21.18	108463.98	85	155.12	29.24	149731.29
3	31.03	2.87	14672.55	86	156.13	14.24	72902.55
4	39.03	23.25	119048.10	87	157.13	45.01	230483.38
5	40.04	7.83	40088.47	88	158.14	16.61	85049.95
6	41.05	90.58	463789.86	89	159.15	24.57	125797.38
7	42.05	6.61	33849.35	90	160.12	8.70	44542.20
8	43.03	37.04	189653.34	91	161.17	20.54	105146.81
9	43.06	12.45	63758.81	92	163.11	24.64	126139.99
10	44.00	3.82	19540.20	93	164.12	9.03	46212.52
11	45.01	4.61	23608.27	94	165.11	7.95	40721.53
12	45.04	3.04	15546.07	95	167.12	9.00	46083.67
13	51.04	5.93	30354.96	96	168.13	4.86	24910.37
14	52.04	3.66	18725.85	97	169.14	27.09	138708.38
15	53.05	44.08	225710.93	98	170.10	4.52	23157.42
16	54.06	5.88	30114.55	99	170.14	9.33	47780.04
17	55.03	21.88	112037.96	100	171.15	42.43	217252.28
18	55.07	47.41	242744.55	101	172.12	13.24	67774.60
19	57.05	5.77	29530.08	102	173.13	21.55	110335.75
20	65.05	24.53	125604.19	103	174.14	7.01	35871.33
21	66.06	9.12	46684.66	104	175.11	5.92	30317.80
22	67.07	43.71	223826.58	105	175.15	4.65	23819.02
23	68.04	5.07	25984.53	106	176.12	17.57	89951.33
24	68.08	11.38	58277.88	107	177.13	6.55	33514.14
25	69.05	12.07	61808.91	108	179.12	4.85	24841.77
26	69.09	10.29	52705.20	109	181.14	8.84	45255.03
27	71.06	4.25	21783.00	110	183.12	48.60	248855.28
28	77.06	54.13	277146.92	111	184.13	19.22	98391.16
29	78.06	16.38	83882.04	112	185.17	36.79	188396.34
30	79.07	67.05	343323.17	113	186.16	11.60	59380.41
31	80.08	11.60	59417.03	114	187.15	10.54	53975.46
32	81.05	3.70	18921.49	115	189.13	14.26	72994.85
33	81.09	60.89	311769.87	116	190.14	6.76	34600.65
34	82.09	5.10	26116.42	117	195.16	9.50	48633.69
35	83.03	3.89	19940.43	118	197.14	37.70	193038.93
36	83.07	4.48	22921.92	119	198.14	17.49	89535.27
37	89.06	3.42	17507.47	120	199.15	18.38	94086.63
38	91.07	100.00	512035.48	121	200.16	8.84	45289.42
39	92.08	16.48	84402.12	122	201.13	25.41	130084.44
40	93.09	28.88	147854.22	123	202.14	15.58	79796.57
41	94.10	4.72	24163.73	124	203.15	10.40	53270.69
42	95.07	6.82	34920.63	125	209.14	3.35	17143.44

Peak	Fragmentation pattern (m/z)	Relative intensity (%)	Intensity	Peak	Fragmentation pattern (m/z)	Relative intensity (%)	Intensity
43	95.11	7.44	38094.92	126	209.17	6.10	31243.79
44	97.05	8.09	41439.53	127	211.19	23.59	120769.47
45	97.09	4.18	21413.02	128	213.16	7.39	37834.50
46	98.06	4.96	25405.56	129	213.20	5.82	29817.68
47	103.08	17.42	89220.66	130	215.15	14.98	76705.22
48	104.08	15.13	77448.59	131	216.16	17.14	87760.43
49	105.09	58.27	298384.27	132	217.17	9.28	47530.58
50	106.10	9.54	48833.50	133	223.16	8.54	43741.62
51	107.07	5.81	29736.56	134	225.21	15.26	78153.66
52	107.11	10.68	54698.20	135	226.17	21.33	109234.81
53	109.09	4.64	23751.78	136	227.19	14.69	75221.90
54	111.07	5.53	28302.59	137	229.17	13.37	68447.53
55	115.08	46.10	236057.94	138	231.18	8.01	41006.01
56	116.09	18.63	95398.13	139	232.19	7.88	40371.66
57	117.10	51.57	264037.45	140	237.18	6.73	34466.45
58	118.10	14.87	76162.78	141	239.19	52.13	266913.86
59	119.11	28.07	143740.50	142	240.20	10.69	54741.98
60	120.12	4.46	22811.48	143	241.18	7.64	39117.49
61	121.09	14.64	74984.38	144	243.17	11.35	58091.09
62	121.13	3.43	17547.24	145	244.17	21.20	108542.62
63	122.10	6.27	32124.70	146	251.19	8.80	45059.77
64	123.11	4.46	22821.91	147	253.21	11.24	57559.82
65	127.08	12.18	62373.54	148	255.19	8.92	45690.92
66	128.09	43.37	222054.34	149	256.20	9.77	50035.95
67	129.10	59.83	306335.36	150	257.21	57.09	292340.22
68	130.10	20.88	106927.74	151	258.21	13.14	67287.28
69	131.11	42.69	218591.83	152	262.17	10.36	53047.31
70	132.12	10.83	55431.24	153	266.22	10.87	55681.63
71	133.09	6.28	32145.29	154	267.23	5.59	28644.31
72	133.13	10.22	52319.58	155	268.24	6.90	35352.70
73	135.11	6.59	33763.51	156	269.21	8.55	43799.24
74	141.10	29.11	149058.13	157	270.21	6.89	35261.99
75	142.11	24.58	125872.34	158	271.22	6.54	33512.39
76	143.12	52.61	269401.06	159	284.23	14.14	72416.01
77	144.12	19.96	102202.93	160	285.24	13.61	69702.70
78	145.13	36.27	185713.95	161	294.22	9.80	50185.53
79	146.10	5.43	27808.54	162	297.21	5.41	27686.34
80	146.14	5.82	29780.10	163	312.24	21.54	110307.30
81	147.11	12.65	64796.96	164	313.24	4.65	23824.56
82	152.09	6.21	31784.72	165	330.25	14.86	76102.35
83	153.10	9.90	50697.82				

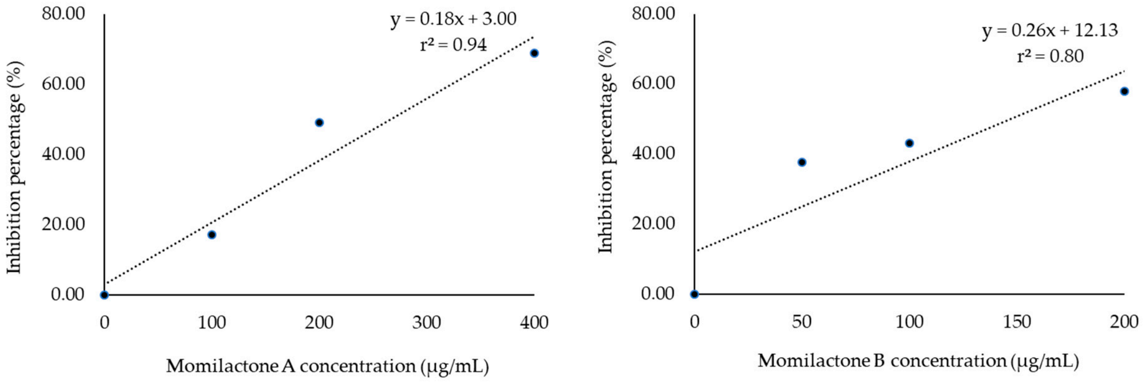

Figure S1. Inhibition of momilactones A and B on α-amylase activity

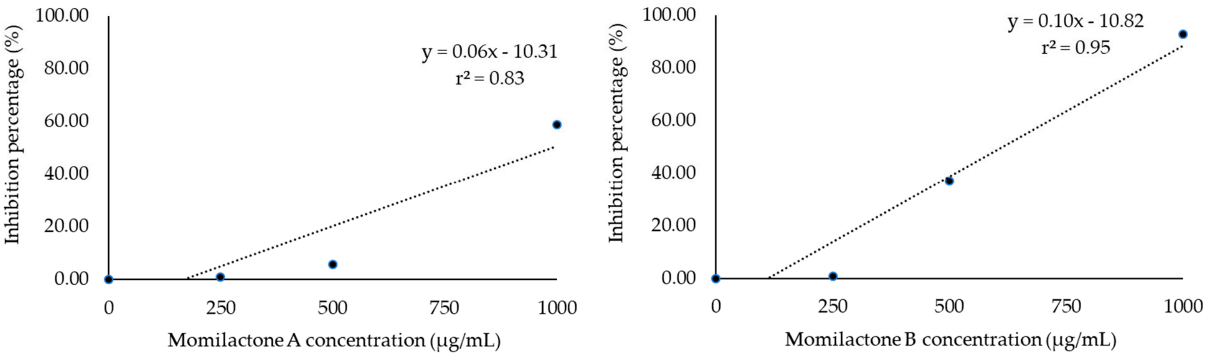

Figure S2. Inhibition of momilactones A and B on α-glucosidase activity
